# An adenovirus-based mucosal vaccine induces cross-neutralizing antibodies and protects against lethal H5N1 infection in ferrets

**DOI:** 10.3389/fimmu.2026.1863888

**Published:** 2026-10-08

**Authors:** Becca A. Flitter, Kayan Tam, Laura J. Showalter, William Sun, Sean N. Tucker

**Affiliations:** Vaxart, Inc., South San Francisco, CA, United States

**Keywords:** adenovirus vaccine, H5N1 clade 2.3.4.4b, IgA, influenza, mucosal immunity, mucosal vaccine, oral vaccine

## Abstract

**Background:**

H5N1 highly pathogenic avian influenza (HPAI) has been detected in multiple animal species, including humans, posing a significant threat to public health. Vaccines capable of eliciting robust mucosal and systemic immunity are needed to protect against HPAI infection.

**Methods:**

First- and second-generation non-replicating adenovirus type-5 vectored vaccines expressing hemagglutinin (HA) from clade 2.1.3.2 (ND1.1) and clade 2.3.4.4b (VXA-H5-02), were administered via peroral route to ferrets. Mucosal and systemic immunogenicity were evaluated. Protective efficacy, viral burden, and shedding was assessed against clade 2.3.4.4b challenge, for clade-matched VXA-H5–02 and clade-mismatched ND1.1 vaccines.

**Results:**

Immunization elicited mucosal immunity and serum neutralizing activity in ferrets against homologous and heterologous clades. All control animals rapidly succumbed to the lethal 2.3.4.4b clade challenge, compared with survival rates of 38% and 100% in the ND1.1 and VXA-H5–02 vaccine groups, respectively. VXA-H5–02 immunization significantly reduced illness, viral burden, and shedding.

**Conclusion:**

Second-generation VXA-H5–02 is a promising vaccine candidate capable of inducing systemic and mucosal immunity, offering broad protection against HPAI.

## 1 Introduction

Emerging in 2020, H5N1 clade 2.3.4.4b HPAI has demonstrated an exceptional ability to infect multiple species, including humans (1). Following its introduction into North America in 2024, the virus caused devastating outbreaks in poultry and cattle, resulting in multiple zoonotic infections in humans. Although the current risk of human-to-human transmission remains low, proximity to farm animals remains a major risk factor in contracting avian influenza. As of May 2026, 71 confirmed cases of H5 infections and two deaths in humans have been reported to the CDC in the United States since 2024 (2). Most cases were associated with exposure to poultry or dairy farms (2).

Vaccination remains a critical intervention for controlling influenza outbreaks and preventing disease spread. Currently, there are three FDA licensed injectable vaccines against H5N1 based on clade 1 (A/Vietnam/1204/2004), clade 2.1.3.2 (A/Indonesia/5/2005), and clade 2.2.1 (A/turkey/Turkey/1/2005 NIBRG-23) influenza strains from prior outbreaks (3). These injected vaccines have demonstrated some cross-reactive and neutralizing potential to clade 2.3.4.4b H5N1 viruses (4). However, cross-protection may be incomplete due to the antigenic drift of H5N1 viruses over time (5). Therefore, vaccine platforms amenable to rapid update of target antigen(s) are essential to ensure optimal protection should a new strain arise.

Effective protection against influenza infection is mediated by immunity at mucosal surfaces, particularly IgA in the respiratory tract, which is sub-optimally and inconsistently elicited by conventional injected vaccines (6, 7). Recent preclinical H5 vaccine candidates, including intranasal live-attenuated, viral-vectored, and RNA-based platforms, have demonstrated protection against HPAI challenge in animal models such as mice, hamsters, and ferrets (8–10). Several of these preclinical candidates require repeated dosing to achieve protective immunity and have yet to demonstrate robust translational mucosal responses or protective efficacy in humans. Furthermore, in the context of pandemic preparedness, many of these candidates may require large investments for cold-chain transport and storage, trained personnel to administer, and extensive healthcare infrastructure for distribution (11). These hurdles highlight the need for temperature-stable next-generation mucosal vaccines that can be rapidly updated, manufactured, and distributed (12).

We have developed an oral tablet vaccine platform that utilizes a non-replicating (E1/E3-deleted) human recombinant adenovirus type 5 vector (rAd5). This platform encodes both a target antigen of interest (i.e., hemagglutinin or HA) and a dsRNA molecular adjuvant designed to enhance protective immunity to the target antigen (11, 13). Packaged in a temperature stable, self-administrated oral tablet, this mucosal vaccine offers multiple practical advantages for pandemic responsiveness. Target antigens can be selected and manufactured rapidly under GMP conditions allowing for vaccines to be modified quickly as new viruses emerge. The oral tablet is thermostable at 30˚C for over two years, reducing the significant costs associated with cold-chain transport, storage, and medical waste disposal (11). Further, the simplicity of oral self-administration may speed distribution to the population and decrease the burden on healthcare infrastructure and personnel needed to administer needle-based vaccines, particularly in a pandemic setting.

We previously demonstrated in a Phase 2b human H1N1 challenge study that a first-generation oral rAd5-H1 tablet approach elicited protective immunity with comparable efficacy to that of a commercial injected influenza vaccine (11, 14–16). This protection was likely mediated by the ability of the oral rAd5-H1 tablet vaccine to induce both systemic and mucosal immune responses. Injected vaccines predominantly elicit systemic responses with limited mucosal immunity, which may contribute to why influenza vaccine effectiveness rarely exceeds 80% across seasons (11, 17, 18).

Other controlled human influenza challenge models have also shown the importance of mucosal immunity where higher pre-existing nasal IgA to H1N1 correlated with limiting infection and infectious shedding (19). The COVID-19 pandemic has renewed interest in mucosal influenza vaccine strategies, as it highlighted the limitations of systemic immunity in preventing respiratory virus infection and transmission (18, 20, 21). Given that mucosal responses play a critical role in protecting against pathogen entry, eliciting immunity in the respiratory tract may be a key requirement for improving future influenza vaccine efficacy (18, 20).

Our first-generation rAd5-H5 vaccine clinical candidate (ND1.1) utilized an antigen based on the HA sequence from H5 clade 2.1.3.2 (22). Ferrets perorally immunized with ND1.1 were protected against a lethal challenge of the homologous H5N1 clade 2.1.3.2 virus (22). Furthermore, ND1.1 in oral tablet form was evaluated in the clinic in a Phase I study, where the vaccine was well tolerated in humans and induced antigen-specific T cell responses (23). Building on our previous findings, a second-generation vaccination platform was developed that increased antigen expression and immunogenicity (24, 25). This second-generation rAd5-H5 approach was used to generate an optimized HPAI vaccine with the goal of enhancing antigen expression, immunogenicity, and manufacturing capabilities.

In this study, we compared the immunogenicity and efficacy of two rAd5-H5 mucosal vaccine candidates, first-generation ND1.1 encoding a clade 2.1.3.2 H5 HA and second-generation VXA-H5–02 encoding a clade 2.3.4.4b H5 HA. Both vaccine candidates were immunogenic and developed cross-reactive serum and mucosal antibodies to multiple clades of H5, with the second-generation VXA-H5–02 demonstrating improved immune responses compared to first-generation ND1.1 vaccinated animals. We further evaluated the protective efficacy of ND1.1 and VXA-H5-02, including their effects on clinical symptoms, using a lethal H5N1 clade 2.3.4.4b challenge that was antigenically mismatched to ND1.1 and matched to VXA-H5-02. Notably, all VXA-H5–02 immunized ferrets survived lethal challenge and exhibited significant reduction in upper respiratory viral burden and shedding post infection. These results highlight the potential of a second-generation mucosal avian influenza vaccine for inducing immunity that protects against disease and impacts viral shedding.

## 2 Results

### 2.1 Second-generation H5 vaccine improved antigen expression *in vitro*

Building on the development of the first-generation rAd5-H5 vaccine (ND1.1) (22), we designed and optimized a second-generation rAd5-H5 vaccine (VXA-H5-02) to streamline vector manufacturing, enhance antigen expression, and improve immunogenicity (**Figure 1A**). Both first- and second- generation rAd5-H5 vaccines utilize a non-replicating rAd5 (E1/E3 deletion), with the antigen-encoding transgene inserted into the E1 region under the control of a CMV promoter. Additionally, a *tet* operator sequence (*tetO*) was included in place of an intron sequence in the second-generation backbone VXA-H5–02 to suppress transgene expression during manufacturing, improving manufacturing yields and consistency. The promoter for dsRNA molecular adjuvant for VXA-H5–02 was updated to be under the control of a SV40 promoter, whereas a CMV promotor was utilized for the ND1.1 first-generation vaccine. Lastly, new sequence optimization methods were utilized when designing the second-generation vaccine to increase target antigen protein production *in vivo.*

**Figure 1:**
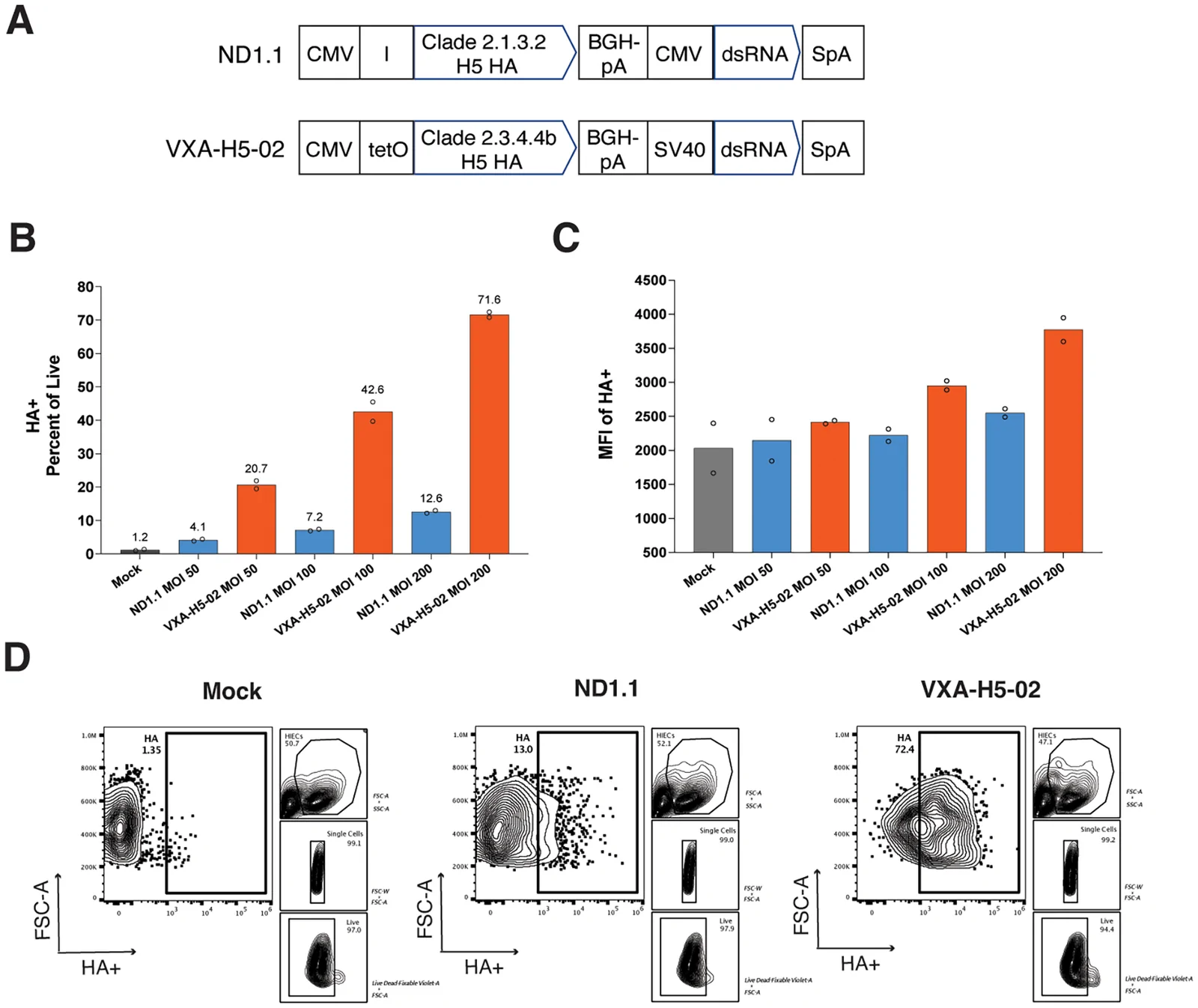
VXA-H5.02 expresses higher levels of H5 in a human intestinal epithelial cell line. **(A)** Schematic depicting the design of ND1.1 and VXA-H5-02. I, intron; *tetO*, tet operator; BGH-pA, bovine growth hormone polyadenylation; CMV, cytomegalovirus promoter; SV40, simian virus 40 promoter. **(B)** 3.7x10^5^ HIEC-6 cells transduced with ND1.1 (blue bars) or VXA-H5-02 (orange bars) at increasing multiplicities of infection (MOI 50, 100, and 200) or mock negative control. At 42 hours post-transduction, cells were harvested, stained for surface expression of HA and analyzed by flow cytometry. Bars indicate mean percent of infected cells across two independent experiments, with symbols representing individual values. **(C)** Median Fluorescence Intensity (MFI) of HIEC-6 cells positive for HA transduced with ND1.1 (blue bars) or VXA-H5-02 (orange bars) across two independent experiments; symbols indicate each individual replicate. **(D)** Representative flow cytometry plots are shown, gated on live, single cells, with the percentage of HA-positive cells indicated.

To compare the surface expression of the HA protein antigen, human intestinal epithelial cells (HIEC-6) were treated with either the first- or second-generation H5 vaccine candidate, ND1.1 or VXA-H5-02, at increasing MOIs (**Figure 1A**). HA expression was measured by flow cytometry using a pan-influenza antibody targeting the conserved HA stem region (26). Across two titration experiments, a higher percentage of HIEC-6 were positive for HA when transduced with VXA-H5–02 at increasing MOI of 50, 100 or 200 compared to ND1.1 treated cells (**Figures 1B, D**). VXA-H5–02 transduction increased the percent of HA expressing cells by at least 5-fold compared to ND1.1 at all MOIs. Furthermore, the median fluorescence intensity (MFI) of HA+ cells increased when transduced with VXA-H5–02 compared to ND1.1, indicating greater antigen abundance per cell (**Figure 1C**). Therefore, in this *in vitro* human intestinal epithelial cell model, the optimized second-generation vaccine VXA-H5–02 improved H5 surface expression compared to the first-generation vaccine ND1.1 (22).

### 2.2 Second-generation H5 vaccine enhanced serum antibody responses in mice

To rapidly assess if increased antigen abundance observed *in vitro* with the second-generation VXA-H5–02 resulted in improved immunogenicity, anti-H5 HA serum IgG antibodies were quantified following intranasal immunization in mice. Mice were intranasally immunized with first-generation ND1.1 encoding a clade 2.1.3.2 H5 HA or second-generation VXA-H5–02 encoding a clade 2.3.4.4b H5 HA (**Supplementary Figure 1A**), and immune responses were tested against homologous and heterologous HAs. Mice intranasally vaccinated with VXA-H5–02 produced higher levels of serum IgG antibodies to three clades of H5 HA tested, clade 2.3.4.4b (A/Greylag Goose/Scotland/024915/2022), clade 2.1.3.2 (A/Indonesia/5/2005), and clade 1 (A/Vietnam/1203/2004) compared to ND1.1 (**Supplementary Figures 1B, C**). This result provided additional *in vivo* evidence, demonstrating that the optimized second-generation vaccine candidate VXA-H5–02 enhanced humoral immunogenicity responses compared to the first generation ND1.1. Together, these results supported further head-to-head testing of ND1.1 and VXA-H5–02 candidates in a ferret H5N1 challenge model to further assess the antigen-specific mucosal responses and impact of homologous and heterologous vaccine administration on protective efficacy.

### 2.3 Perorally immunized ferrets produced systemic and mucosal cross-reactive antibodies against H5 HA

Ferrets are commonly used to evaluate influenza vaccine immunogenicity and efficacy in preclinical studies. Influenza naturally infects the upper respiratory tract in ferrets and they display similar clinical symptoms to humans, including fever, sneezing, coughing, weight loss, and lethargy (27). To model oral administration of vaccine tablets developed for clinical use, the vaccines were delivered directly to the small intestine perorally by endoscopy in a liquid formulation. Ferrets received 1 x 10^10^ infectious unit (IU) of ND1.1 or VXA-H5–02 diluted in PBS, administered by endoscopy to the pyloric sphincter on Day 0 and Day 28 (**Figure 2A**). This vaccine prime and boost schedule was determined in previous ferret challenge studies conducted with ND1.1 comparing injected vaccine to peroral administration (22).

**Figure 2:**
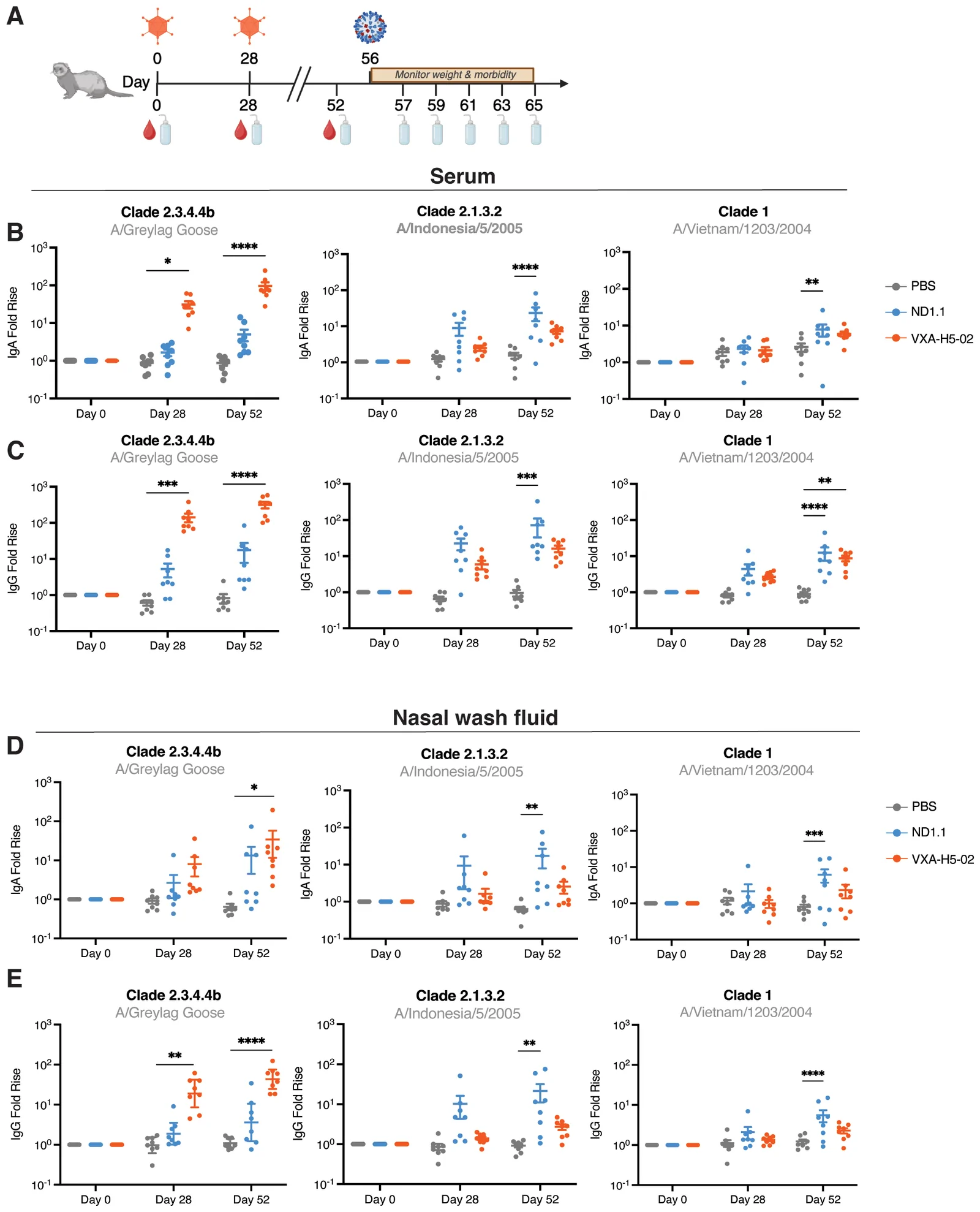
Ferrets perorally vaccinated via the endoscopy generates cross-reactive antibodies. **(A)** Schematic of study design (n = 8 per group). Male and female ferrets were immunized by endoscopy with PBS, 1 x 10^10^ IU of ND1.1 or 1 x 10^10^ IU of VXA-H5–02 directly to the small intestine on Day 0 and 28. Serum and nasal wash fluid (NWF) samples were collected on Day 0, 28, and 52 to measure antibody response. **(B–E)** Fold rise of serum IgA **(B)**, serum IgG **(C)**, nasal wash fluid IgA **(D)**, nasal wash fluid IgG **(E)** of anti-clade 2.3.4.4b HA (left), anti-clade 2.1.3.2 HA (middle), and anti-clade 1 HA (right) of ND1.1 or VXA-H5–02 immunized ferrets. Graphs representing the average of 8 ferrets (4M/4F) per group +/- S.E.M. Statistical analysis was performed using a 2-way ANOVA with Dunnett’s multiple comparison test. *p < 0.05, **p < 0.01, ***p < 0.001, ****p < 0.0001.

Ferrets perorally vaccinated with VXA-H5–02 produced robust systemic IgG and IgA responses detectable in the serum on Day 28 and Day 52 post-vaccination (**Figures 2B, C**; **Supplementary Figure 2**). Notably, VXA-H5–02 or ND1.1 perorally vaccinated ferrets developed robust IgG and IgA mucosal antibody responses targeting the matched H5 HA in nasal wash fluid (NWF) (**Figures 2D, E**; **Supplementary Figure 2**). Antibody cross-reactivity was observed in both ND1.1 and VXA-H5–02 vaccinated ferrets to different H5 HAs in both serum and NWF (**Figures 2B–E**). Overall, these results demonstrated that peroral vaccination induces broad and robust systemic and upper respiratory antibody responses in ferrets.

### 2.4 VXA-H5–02 immunized ferrets produced neutralizing antibodies against H5N1 pseudoviruses

To test the neutralization potential of vaccine-induced antibodies, serum collected on Day 52 from immunized ferrets was preincubated with alpha-pseudovirus particles assembled from clade 2.3.4.4b (A/Nevada/10/2025, Genotype D) influenza structural proteins (HA, neuraminidases, nucleoprotein, matrix protein M1, and the ion channel M2). The mixture was incubated with luciferase reporter cells and percent infection was quantified. Serum from VXA-H5–02 immunized ferrets produced potent clade matching 2.3.4.4b-serum neutralizing antibodies and had a median 50% neutralizing titer of 1:125 (**Figure 3A**). ND1.1 immunized ferret serum exhibited only 57% infection at the highest serum dilution (1:5) assessed, therefore the 50% neutralizing titer could not be determined to the heterologous pseudovirus (**Figure 3A**). Although ND1.1 immunized animals produced weak serum functional responses to 2.3.4.4b, this result was not unexpected utilizing the first-generation rAd5-H5 approach against a mismatching pseudovirus. Lastly, no neutralizing antibodies were detected in the serum from the PBS control group (**Figure 3A**).

**Figure 3:**
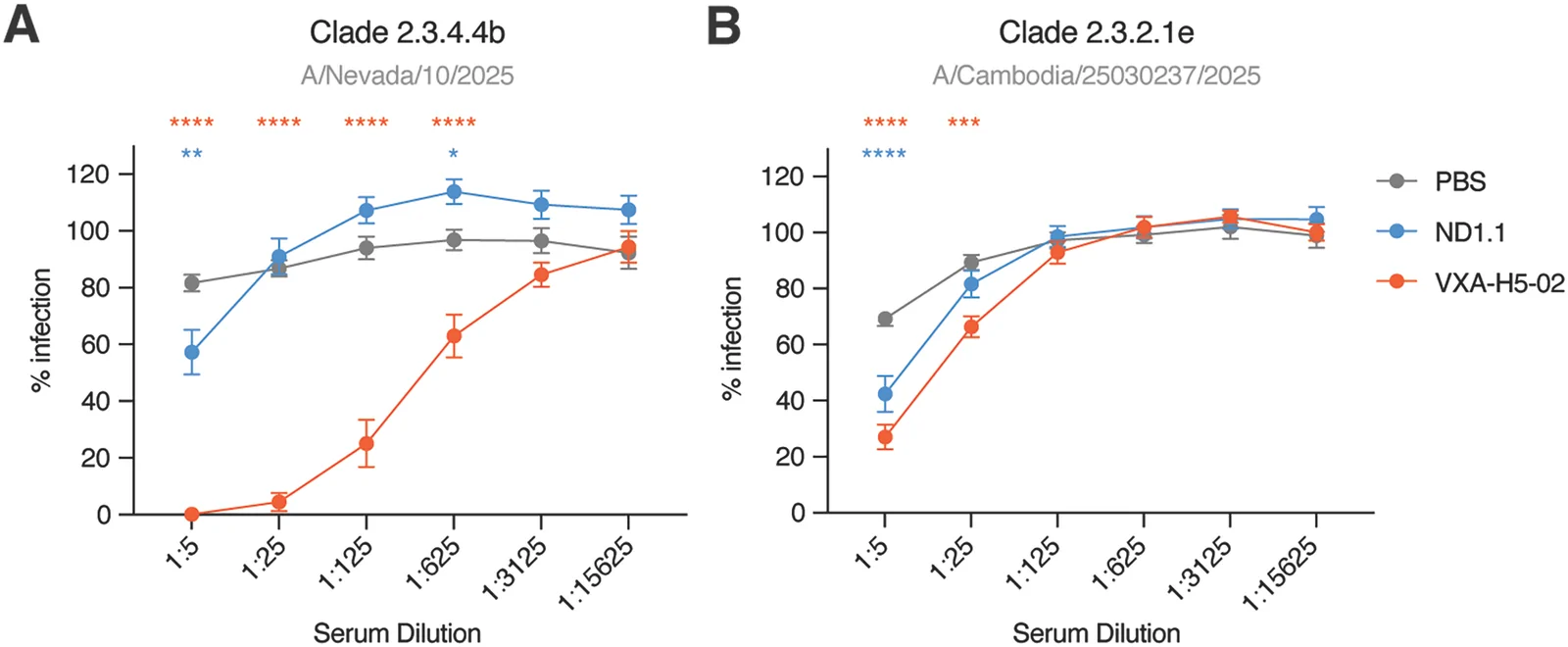
Ferrets perorally vaccinated with VXA-H5–02 produce cross-neutralizing antibodies in serum. **(A, B)** Serial dilution of serum collected on Day 52 from PBS, ND1.1, or VXA-H5–02 groups neutralized alpha-pseudovirus for **(A)** clade 2.3.4.4b virus (Genotype D, A/Nevada/10/2025) or **(B)** clade 2.3.2.1e virus (A/Cambodia/25030237/2025). Percent infected cells were determined by measuring the luminescence at 20 hours post infection. Graph represents the average of 8 ferrets per group +/- S.E.M. Statistical analysis was performed using a 2-way ANOVA corrected with Dunnett’s multiple comparisons test. * p < 0.05, **, p < 0.01, **** p < 0.0001.

To assess functional activity in mucosal secretions, NWF from all three groups on Day 52 was also evaluated for neutralization against 2.3.4.4b alpha-pseudovirus. Interestingly, neutralizing activity to 2.3.4.4b alpha-pseudovirus in the NWF was observed in all groups; however, a higher number of VXA-H5–02 immunized ferrets elicited mucosal functional activity (**Supplementary Figure 3A**). This was likely driven by greater numbers of VXA-H5-02-immunized ferrets generating antigen matched specific neutralizing antibodies in the NWF (5/8 ferrets), while PBS and ND1.1 groups had 2/8 and 3/8 ferrets, respectively (**Supplementary Figure 3B**). A key consideration is the lower abundance of antibody that can be collected in the NWF, which is considerably more dilute than serum, rendering this sample type difficult to access for functional assays. Therefore, the observation that many of the VXA-H5–02 immunized ferrets had detectable neutralizing activity in the NWF demonstrates the enhanced ability of the second-generation vaccine to generate robust functional mucosal responses.

The recent emergence of H5N1 clade 2.3.2.1e (also known as clade 2.3.2.1c) in Cambodia highlights the danger of influenza reassortment and the lethality of HPAI (28, 29). H5 HAs belonging to different clades share a high degree of structure homology and protein sequence identity. Using the Clustal Omega protein sequence alignment tool, multiple H5 HA sequences were aligned: Clade 2.3.4.4b (represented by the VXA-H5–02 transgene), clade 2.1.3.2 (represented by the ND1.1 transgene), clade 1 (represented by A/Vietnam/1203/2004), and recently emerged clade 2.3.2.1e. The HA protein sequences of VXA-H5-02, ND1.1, and A/Vietnam/1203/2004 (another licensed H5 vaccine) shared 92-96% protein sequence identity. Protein sequence alignment shows that most differences were localized to the head domain, consistent with its role as an immunodominant target under continuous immune selection pressure (**Supplementary Figure 4**). Many of these differences are also clustered within known antigenic sites (30). These findings provide a molecular basis for evaluating vaccine induced cross-reactive immune responses across distinct viral clades.

The HA of clade 2.3.2.1e and clade 2.3.4.4b share similarity with several amino acids in the head domain, suggesting cross-neutralizing antibodies may be possible in the ND1.1 and VXA-H5–02 immunized ferrets (**Supplementary Figure 4**). To assess this, serum collected on Day 52 from immunized ferrets was preincubated with alpha-pseudovirus particles assembled from clade 2.3.2.1e (A/Cambodia/25030237/2025). Detectable neutralizing activity was observed following immunization with either ND1.1 or VXA-H5–02 against heterologous clade 2.3.2.1e. At 1:5 serum dilution, animals immunized with either VXA-H5–02 and ND1.1 produced cross-functional antibody responses in the serum that significantly impacted clade 2.3.2.1e alpha-pseudovirus infection compared to PBS control serum (**Figure 3B**). At 1:25 serum dilution, only animals vaccinated with VXA-H5–02 produced significantly more functional antibody against 2.3.2.1e alpha-pseudovirus compared to PBS. (**Figure 3B**). Although both vaccines demonstrated functional responses to clade 2.3.2.1e at a low dilution, VXA-H5–02 immunization induced slight increased cross-neutralizing activity, most likely due to improvements with second-generation vaccine design enhancing antigen expression. Altogether, these results demonstrated the potential of the rAd5 avian influenza mucosal vaccine to elicit both antigen-matched neutralizing antibodies and cross-functional responses to heterologous influenza clades.

### 2.5 VXA-H5–02 immunized ferrets were protected from lethal clade 2.3.4.4b H5N1 infection

On Day 56 post-vaccination, ferrets were intranasally challenged with 1 x 10^5^ TCID_50_ of A/dairy cattle/Texas/24-008749-002-v/2024, a genotype B, clade 2.3.4.4b virus (**Figure 2A**). To assess protective efficacy, survival was monitored for nine days following viral challenge and analyzed by Kaplan-Meier (**Figure 4A**). All control animals perorally administered PBS succumbed to lethal clade 2.3.4.4b viral challenge (n=0/8) by Day 6 post infection. ND1.1 vaccination resulted in 38% survival nine days post infection (n=3/8), a meaningful result following immunization with first-generation vector backbone against a mismatched clade challenge. In contrast,

**Figure 4:**
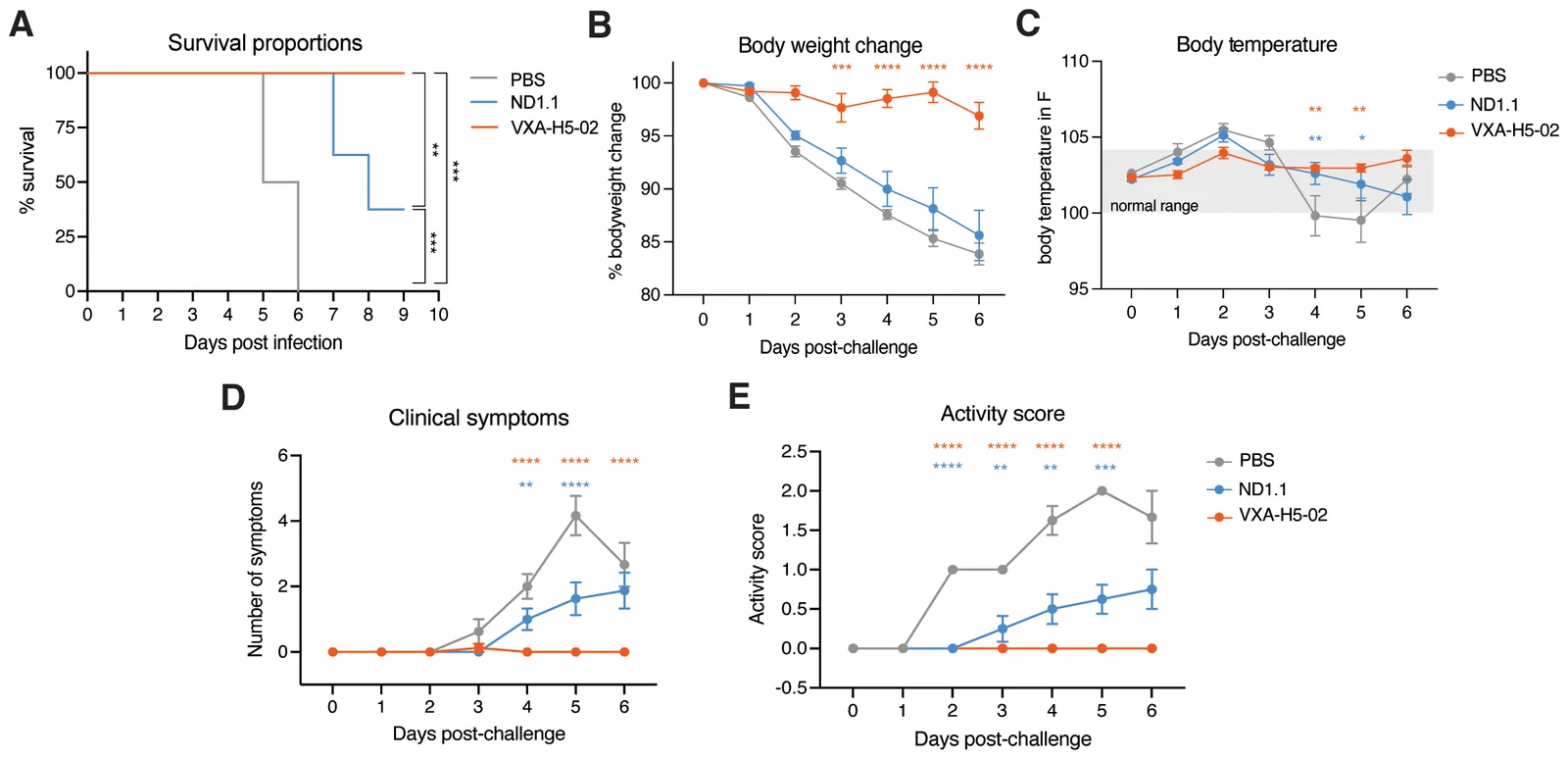
Ferrets perorally vaccinated with VXA-H5–02 are protected from lethal clade 2.3.4.4b infection. **(A)** Kaplan-Meier curves depicting % survival of immunized ferrets challenged with 1 x 10^5^ TCID_50_/ml of clade 2.3.4.4b virus (Genotype B, A/Texas/Dairy Cow/2024). Log-rank Mantel-Cox test followed p-value adjusted for multiple comparisons with Holm-Sidak correction shown, relative to the PBS control group ND1.1 (p < 0.001) and VXA-H5-02 (p < 0.001) and ND1.1 and VXA-H5–02 relative to each other (p = 0.009). Ferrets were evaluated for **(B)** % body weight change **(C)** body temperature **(D)** number of clinical symptoms (i.e. dehydration, diarrhea, nasal discharges, hypoactivity, sneezing, rough coat, abnormal breathing) **(E)** activity post-infection. Statistical analyses were performed using a Mixed Effect analysis, with Dunnett’s multiple comparisons test. *p < 0.05, **p < 0.01, ***p < 0.001, ****p < 0.0001.

100% of VXA-H5–02 vaccinated ferrets survived through Day 9 post-challenge (n=8/8), demonstrating superior efficacy to ND1.1. A log-rank (Mantel-Cox) test revealed a highly significant difference in overall survival outcomes across the three experimental groups (p = <0.0001). *Post-hoc* pairwise comparisons with Holm-Sidak correction were subsequently conducted to determine differences between individual groups. These comparisons revealed survival was significantly improved in both ND1.1 (p < 0.001) and VXA-H5-02 (p < 0.001) groups relative to the PBS control group (**Figure 4A**). Moreover, survival in the ND1.1 group compared to VXA-H5–02 group was found to be significantly different (p = 0.009). Survival proportions across the three experimental groups were further analyzed by two-sided Fisher’s exact test. VXA-H5–02 vaccinated animals showed a highly significant survival benefit compared to the PBS group (p < 0.001; odds ratio = 0.00, 95% CI [0.00, 0.120]). Although ND1.1 vaccination resulted in numerical improvement compared to the PBS group, this difference was not statistically significant (p = 0.200; odds ratio = 0.00, 95% CI [0.00, 2.205]). Lastly, the VXA-H5–02 group demonstrated a significant survival advantage over ND1.1 (p = 0.026; odds ratio = ∞, 95% CI [1.33, ∞]).

During the challenge period, clinical symptoms were assessed daily for all ferrets. ND1.1-vaccinated ferrets lost weight during the first 7 days post-infection (**Figure 4B**; **Supplementary Figure 5A**) and experienced fever (**Figure 4C**; **Supplementary Figure 5B**). In contrast, VXA-H5-02-vaccinated ferrets showed only minor weight loss (**Figure 4B**; **Supplementary Figure 5A**), losing less than 10% of body weight throughout the challenge period. Six of eight VXA-H5-02-vaccinated ferrets developed a brief fever that resolved within two days (**Figure 4C**; **Supplementary Figure 5B**).

Clinical signs were recorded for each animal as the total number of observed symptoms, independent of severity (**Supplementary Figure 6**). Classical symptoms associated with influenza infections in ferrets include dehydration, diarrhea, eye/nasal discharges, hypoactivity, sneezing, rough coat, and abnormal breathing (31). The complete list of documented symptoms after influenza challenge is listed for each ferret (**Supplementary Figure 6**). Signs of infection first appeared in the PBS control group on Day 3 post-challenge and peaked on Day 5. ND1.1-vaccinated animals developed symptoms on Day 4, with peak symptoms on Day 7, but overall showed fewer symptoms than PBS controls. VXA-H5-02-vaccinated animals had few or no influenza-associated clinical signs (**Figure 4D**). In this group, one of eight animals showed one symptom on Days 3 and 9 post-challenge, and three of eight animals showed one symptom late in the challenge period, on Days 7 and 8 (**Figure 4D**; **Supplementary Figure 5C**).

Activity was scored daily after challenge using a four-point scale used by Reuman et al. (32), 0 = alert, playful, and active; 1 = alert, but playful or active only when stimulated; 2 = alert, but not playful or active when stimulated; and 3 = neither alert nor playful or active when stimulated. PBS control animals had the highest activity scores, peaking on Day 5 post-challenge, whereas ND1.1-vaccinated animals showed delayed onset and lower peak activity scores on Day 7. All VXA-H5-02-vaccinated ferrets maintained normal activity scores throughout the study (**Figure 4E**; **Supplementary Figures 5C**, **6**).

Together, these findings demonstrate that antigen-matched VXA-H5–02 was highly protective against lethal clade 2.3.4.4b infection. While ND1.1 provided moderate protection against the mismatched clade 2.3.4.4b challenge, VXA-H5–02 was significantly more protective.

### 2.6 VXA-H5–02 and ND1.1 vaccination reduced H5N1 viral load and shedding in upper respiratory tract

To evaluate viral burden in the nasal mucosa, NWF were collected from all infected ferrets on Days 1, 3, 5, 7, and 9 post infection (Day 57, Day 59, Day 61, Day 63, and Day 65 post-vaccination, respectively) (**Figure 5A**). Due to disease severity with the lethal challenge, some ferrets in the PBS and ND1.1 groups had their NWF collected one day earlier on Day 6 or Day 8, these samples were included in Day 7 or Day 9 analyses, respectively. The number of viral RNA copies in each sample was determined by RT-qPCR. PBS and ND1.1 vaccinated ferrets demonstrated similar levels of viral load through Day 5 post infection, though the ND1.1 group trended toward lower levels of viral RNA by Day 6 (**Figure 5A**). In contrast, VXA-H5–02 vaccinated ferrets had a significantly reduced viral load in the NWF compared to both ND1.1 and PBS vaccinated ferrets at all time points (**Figure 5A**). Furthermore, seven out of eight VXA-H5–02 immunized ferrets efficiently cleared the virus with no detectable viral load using RT-qPCR by Day 8/9 post challenge (**Figure 5A**).

**Figure 5:**
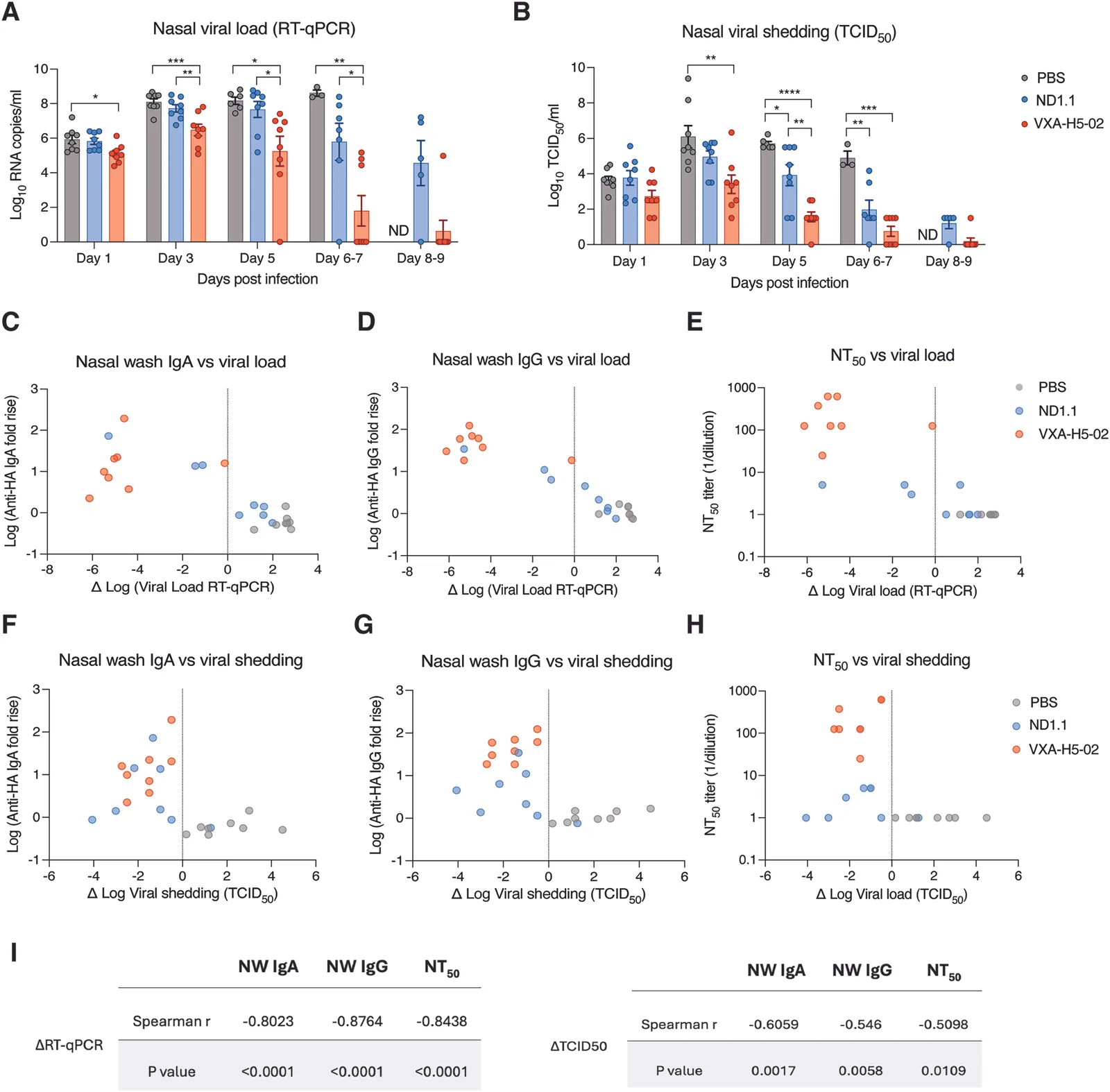
Ferrets perorally vaccinated with VXA-H5–02 have reduced viral load in NWF that correlates to antigen-specific antibody levels. **(A, B)** Viral load of NWF post infection measured by **(A)** RT-qPCR and **(B)** Tissue Culture Infectious Dose 50 (TCID_50_) assay. Each circle represents an individual ferret. Bar represents the average for the indicated group +/- S.E.M. Statistical analyses were performed using a 1-way ANOVA with Tukey’s Multiple Comparisons for Day 1, 3, and 5. For a day’s 6–7 a 1-way ANOVA was conducted with the surviving 3 ferrets in the PBS group *p < 0.05, **p < 0.01, ***p < 0.001, ****p < 0.0001. ND = no data. **(C–E)** Scatter plot comparing change in viral load by RT-qPCR in PBS, ND1.1 and VXA-H5–02 groups to Day 52 anti-clade 2.3.4.4b-specific antibody responses, **(C)** nasal wash fluid (NWF) IgA, **(D)** NWF IgG and **(E)** serum neutralization (NT_50_). Each circle represents an individual ferret. Change in (Δ) viral load was determined by subtracting Day 1 RT-qPCR value from last available RT-qPCR value for each ferret. Dotted line at zero on x-axis indicates no change in viral burden. **(F–H)** Scatter plot comparison of infectious viral shedding by TCID_50_ in PBS, ND1.1 and VXA-H5–02 ferrets to Day 52 anti-clade 2.3.4.4b-specific **(F)** nasal wash fluid (NWF) IgA, **(G)** NWF IgG **(H)** serum neutralization. Each circle represents an individual ferret n=8. Change in (Δ) viral shedding was determined by subtracting Day 1 TCID_50_ value from last available viral TCID_50_ value for each ferret. Dotted line at zero on x-axis same comment (Δ) indicates no change in shedding. **(I)** Spearman r correlation of NWF and NT_50_ antibody responses for all groups compared to viral burden (left) and infectious viral shedding (right).

The nasal wash post-challenge was additionally evaluated by TCID_50_ to determine viable viral particle shedding. No statistical differences were observed for all three groups on Day 1. ND1.1 immunized ferrets began to clear the virus on Day 5, exhibiting a 1.8-log reduction compared to the PBS group. By Day 9, only a low level of viable virus was detected in the ND1.1 NWF samples (**Figure 5B**). VXA-H5–02 immunized ferrets exhibited a significant reduction in viral shedding (a ~2.3 log reduction) compared to the PBS group on Day 3 (**Figure 5B**). The significant reduction in viral shedding continued for the remainder of the challenge period for VXA-H5–02 vaccinated animals. By Day 5, only a low level of viable virus was measured (mean TCID_50_ on Day 5 = 98.5), and samples from Day 6/7 and Day 8/9 were below the detection limit (**Figure 5B**). Collectively, peroral vaccination produced robust mucosal immunity in the VXA-H5–02 vaccinated ferrets that reduced overall viral load and viable virus particles in the NWF. This finding suggests that the delivery of rAd5 to the intestinal mucosa can induce potent distal upper respiratory tract immunity in ferrets to significantly reduce viral load and infectious particle shedding.

We further calculated the change (Δ) in viral load (RT-qPCR) or infectious particle shedding (TCID_50_) by subtracting Day 1 values from the last available sample collected post challenge for each individual ferret. The changes in viral load for individual ferrets were plotted against either nasal antibody fold rise (IgA and IgG) or 50% of serum neutralization titer (NT_50_) on Day 52 for all challenged animals (**Figures 5C–E**). Animals that controlled replication and shedding had viral load changes below zero on the x-axis. In contrast, animals that had productive infection and continued shedding during the challenge period had viral loads and shedding above zero (**Figures 5C–H**). Overall, individual ferrets with higher levels of anti-clade 2.3.4.4b nasal IgA, IgG, and serum NT_50_ on Day 52, prior to challenge, controlled viral burden and particle shedding post-challenge. Conversely, individual animals with less nasal IgG, nasal IgA, or serum NT_50_ showed increases in viral load (**Figures 5C–H**). A Spearman Correlation analysis was conducted on individual animals to further examine the impact of mucosal and systemic clade 2.3.4.4b-specific antibody responses on viral load and infectious particle shedding. Overall, strong antibody-specific responses in the nasal washes and serum demonstrated a strong negative correlation with lower viral loads and shedding (**Figure 5I**). The immune parameter observed with the strongest negative correlation against infectious particle shedding was nasal IgA (-0.6059), whereas nasal IgG and NT_50_ had slightly weaker negative correlations (-0.5460 and -0.5098, respectively) (**Figure 5I**). Together, these results highlight the capacity of this oral vaccine approach to induce strong mucosal and functional systemic HA-specific humoral responses that control influenza burden and shedding following challenge, further corroborating long-standing evidence of the importance of antigen-specific mucosal antibody and serum neutralizing responses as important immune functions for controlling influenza infection (33).

## 3 Discussion

There are currently no approved vaccines specifically targeting clade 2.3.4.4b HPAI, which recently emerged in poultry and dairy cows in the United States. In this study, we demonstrated that a perorally administered second-generation rAd5-H5 vaccine fully protected ferrets from a lethal matched clade 2.3.4.4b challenge. VXA-H5–02 provided full protection against clade 2.3.4.4b challenge, with 100% of immunized ferrets surviving, whereas all PBS control animals succumbed to the infection by Day 6. VXA-H5–02 vaccinated ferrets exhibited minimal clinical symptoms following viral challenge, whereas control animals showed substantial signs of influenza-like illness before death. The ferrets vaccinated with the first-generation antigenically mismatched ND1.1 vaccine (clade 2.1.3.2) exhibited mild symptoms and 38% survived the clade 2.3.4.4b challenge. We also observed significant reductions in live particle viral shedding in the upper respiratory tract of both VXA-H5–02 and ND1.1 immunized ferrets following challenge compared to the control group. Individual ferret viral burden and shedding had the strongest negative correlation with clade 2.3.4.4b-specific nasal antibody levels prior to challenge, indicating vaccine-induced mucosal antibody responses were critical for limiting productive infection in protected animals.

As vaccine administration route, modality, and adjuvants shape the resulting immune response, our findings show that peroral delivery of the rAd5-H5 vaccines induce both systemic and mucosal cross-reactive protective immunity. We observed that ferrets immunized with VXA-H5-02, the second-generation rAd5-H5 vaccine, elicited stronger cross-functional serum neutralizing activity and mucosal antibodies responses compared to ND1.1. This observation is most likely attributable to optimization of the second-generation rAd5-H5 vaccine design, which led to significantly improved *in vitro* H5 HA expression and consequently, enhanced *in vivo* immunogenicity. Although ND1.1 and VXA-H5–02 encode distinct transgene antigens, antigen abundance observed *in vitro* in the HIECs could be directly compared using a monoclonal antibody that recognizes the conserved stem domain of H5 HA. While the second-generation clade 2.3.4.4b vaccine, VXA-H5-02, was antigenically matched to the challenge virus and provided superior protection against severe illness, viral load and shedding, the ferrets immunized with the first-generation clade 2.1.3.2 mismatched vaccine, ND1.1, were still afforded moderate protection against a high viral load challenge. This observation verifies that delivering either first- or second-generation rAd5-H5 vaccines directly to the small intestines, where a high abundance of Peyer’s patches reside, induces functional cross-reactive humoral responses. The partial protection conferred by ND1.1 against a divergent H5 virus highlights the breadth of immune protection that can be achieved with the rAd5 vaccine approach when delivered to the intestinal space.

Other stockpiled injected H5 vaccines have shown limited capacity to elicit cross-protective immune breadth, most likely due to the intramuscular administration route. For example, in a recent H5 preclinical vaccine study, identical injected vaccines differing only in the HA antigen did not produce cross-neutralizing antibodies and were unable to provide any cross-protection in a high viral load clade 2.3.4.4b challenge (5). While weakly cross-neutralizing antibodies to clade 2.3.4.4b have been observed in humans receiving the injected licensed H5 avian influenza (clade 1 or clade 2.1) parenteral vaccines (4), it is unclear if the protection offered by these mismatched vaccine antigens will be sufficient to prevent disease against the latest influenza H5 virus. Injected vaccine approaches may need to be updated more frequently to be antigenically matched to the circulating clades to provide full protection, especially as new pandemic HPAI variants arise that deviate substantially from the vaccine stockpile. Although the oral rAd5 vaccine platform can be rapidly updated and manufactured, as demonstrated here, the induction of cross-clade functional antibodies against divergent H5 clades provides an additional advantage in addressing the ongoing evolution of influenza viruses.

Recent investigational clade 2.3.4.4b-targeting vaccines, such as mRNA-lipid nanoparticles delivered by intramuscular injection (10, 34), or live-attenuated influenza vaccine delivered by intranasal inoculation (35), have also shown effectiveness in protecting against lethal disease in preclinical animal models. Moderna’s avian influenza vaccine, mRNA-1018, was found to be safe and immunogenic in a Phase I/II clinical trial (NCT05972174) (36). While the mRNA or other injected approaches induce potent systemic immune responses, they are poor at eliciting mucosal immune responses, which may provide greater protection against respiratory pathogens (11, 14, 15, 37).

Mucosal immunity is an essential protective immune function, with IgA responses being critical for blocking viral entry and replication at the primary site of infection (19, 37–40). Mucosal IgA is superior for neutralization and cross-protection against divergent influenza virions in the respiratory tract due to the higher avidity and dimeric structure (19, 37, 41). A recent small phase I study investigated two doses (twenty-eight days apart) of an intranasal vaccine comprised of recombinant H5 (A/Indonesia/05/2005 clade 2.1) formulated with nano emulsion adjuvant W_80_5EC, followed by an intramuscular heterologous boost at 6 months with inactivated H5N1(A/Vietnam/1203/2004, clade 1) (NCT05397119) (42). While binding serum antibody increased slightly following two intranasal immunizations, functional responses only reach protective levels following intramuscular vaccination. Furthermore, intranasal immunization elicited moderate dose-independent nasal IgG and IgA and was only detected upon co-administration with W_80_5EC adjuvant.

Clinical findings support the importance of strong mucosal IgA responses for protection against influenza, as baseline nasal IgA against H1N1 was associated with protection against A/California/2009 H1N1 challenge by limiting duration of infection and reducing viral load (19). We have previously demonstrated in a phase 2b A/California/2009 H1N1 challenge study that participants who received oral tablet rAd5-H1 HA influenza vaccine, VXA-A1.1, had increases in mucosal homing HA IgA specific plasmablasts, which correlated with a reduction in symptomatic disease and viral shedding (14, 15). VXA-A1.1 was directly compared in this clinical study to the marketed injected vaccine Fluzone and in a *post-hoc* analysis was more effective at reducing viral shedding (80% probability of superiority based on Bayesian analysis) (11, 15). A smaller proportion of participants immunized with VXA-A1.1 had fewer PCR confirmed infections (36% for VXA-A1.1, 44% for Fluzone, and 71% for placebo) and reported less illness (29% for VXA, 35% for Fluzone compared to 48% for placebo) following influenza challenge (11, 14, 15). Although those participants that received an injected Fluzone vaccine showed stronger serum HAI responses, individuals who received VXA-A1.1 also elicited functional antibody responses following a single oral tablet administration that correlated with protection. These clinical findings demonstrate that oral vaccination can induce both systemic and importantly, mucosal immunity, resulting in protection against influenza infection and disease.

It is plausible to further postulate that strong mucosal responses, particularly IgA, not only impact virus entry and shedding, but may also reduce viral transmission – an important consideration for future influenza outbreak control and containment of H5N1 (19). Beyond influenza, this oral rAd5 vaccine platform has been shown to reduce viral transmission in SARS-CoV-2 preclinical studies. Hamsters vaccinated with an rAd5 encoding the spike protein by oral gavage developed robust mucosal antibody responses and had reduced SARS-CoV-2 viral load, disease burden, and transmission upon challenge (43). While these two respiratory viral pathogens have different transmission kinetics, it is plausible to postulate the vaccine-induced robust IgA antibody responses elicited at multiple barrier surfaces could curtail transmission.

Furthermore, we have previously demonstrated that the first-generation oral tablet rAd5 platform reduces viral shedding in humans against the gastrointestinal viral pathogen, norovirus. In a phase 2b norovirus challenge study, oral tablet administration of rAd5 encoding VP1 capsid protein (VXA-G1.1-NN) was evaluated for illness, infection shedding, and immunogenicity (44). Participants who received VXA-G1.1-NN oral norovirus vaccine tablet generated strong mucosal IgA responses at multiple barrier surfaces, as measured in the nasal lining fluid, saliva, and feces. Furthermore, they had significantly lower norovirus shedding in stool and emesis (44). These results demonstrate that this oral tablet vaccine approach can elicit robust mucosal IgA responses in humans that correlate with reduced shedding of infectious viral particles. Reducing infectious particle shedding could potentially impact transmission dynamics. Additional larger clinical studies are needed to determine whether reductions in viral shedding mediated by this oral vaccine technology translate into reduced transmission.

Several limitations should be considered when interpreting the findings in this study. First is the absence of a direct comparison in the challenge model to a licensed injected H5 vaccine. Parenteral H5 vaccine platforms, including adjuvanted inactivated formulations and emerging mRNA-based candidates, have demonstrated robust hemagglutination inhibition and virus neutralizing antibody responses in humans, with protection primarily associated with systemic immune responses (4, 36). However, because these responses are predominantly restricted to the systemic circulation, they may not provide optimal mucosal immunity at the site of viral entry. In contrast, the oral vaccine evaluated here was designed to induce both systemic and mucosal immune responses, including at the respiratory tract, which may provide complementary mechanisms of protection against infection and viral transmission. Because no injected comparator was included, the relative contributions of these distinct immunological approaches on protection against clade 2.3.4.4b H5 challenge in ferrets could not be assessed. Future head-to-head studies incorporating approved or representative parenteral H5 vaccines will be important for defining the comparative strengths and limitations of oral and injectable vaccination strategies and for positioning this platform within the broader H5 vaccine landscape.

Another limitation of this study is that cellular immune responses were not evaluated, as the primary outcome was systemic and mucosal humoral immunogenicity following vaccination. T cell immunity has been shown to play an important role in protection against influenza viruses in ferrets by promoting viral clearance and recognizing conserved epitopes that may confer cross-strain protection (45). We have also previously demonstrated the contribution of T cell immunity elicited by ND1.1 immunization in humans (23). As the assessment of cellular responses was not included in the present study, the contribution of T cell responses to the protection observed following vaccination and challenge could not be evaluated. Future studies incorporating cellular immune endpoints will be important for a more complete understanding of the mechanisms underlying protection following vaccination with VXA-H5-02.

Lastly, the relatively small number of animals included in each group (n = 8) limited the statistical power of the study and may have reduced the ability to detect modest differences between the two vaccination groups. As a result, some potentially meaningful biological effects may not have reached statistical significance. Despite these limitations, meaningful trends were observed across multiple study endpoints. Future studies with larger cohorts will be important for confirming these findings and providing more precise estimates.

Influenza viruses are constantly evolving, and new variants can arise unpredictably, thus highlighting the urgency for effective control measures to prevent outbreaks. While the risk of HPAI remains low for the public, frequent outbreaks in farm animals and the proximity of farm workers to infected animals increase the risk of humans contracting HPAI. This environment provides opportunities for the virus to cross species barriers. The ability to quickly develop and produce a matching HPAI vaccine that provides mucosal protection in the event of an outbreak is desirable. When formulated as an enteric coated tablet for human use, this oral vaccine is thermostable, reducing reliance on cold-chain infrastructure, and can be self-administered, simplifying large-scale vaccine distribution and administration (11). Collectively, these findings demonstrate that an orally delivered clade 2.3.4.4b vaccine can induce robust serum neutralizing antibodies and cross-reactive mucosal immunity while protecting against lethal disease, supporting its potential as a novel strategy for influenza prevention and pandemic preparedness.

## 4 Materials and methods

### 4.1 Adenovirus vector generation

Non-replicating recombinant adenovirus type 5 (rAd5) vaccine vectors, ND1.1 and VXA-H5-02, were constructed to express H5 hemagglutinin (HA). ND1.1 encoded the HA sequence from A/Indonesia/5/2005 (clade 2.1.3.2; GISAID accession EPI_ISL_121541), whereas VXA-H5–02 encoded the HA sequence from A/England/232540656/2023 (clade 2.3.4.4b; GISAID accession EPI_ISL_17980947). The VXA-H5–02 HA coding sequence was codon optimized to enhance antigen expression. Both vectors were based on a replication-defective rAd5 backbone containing deletions in the E1 and E3 regions, with the HA expression cassette inserted at the E1 locus. In both constructs, HA expression was driven by a cytomegalovirus (CMV) promoter. VXA-H5–02 incorporated a *tet* operator (*tetO*) sequence in place of the intron used in the first-generation vector to suppress transgene expression during vector production and thereby improve manufacturing yield and consistency. Both vectors also encoded a molecular dsRNA adjuvant. Expression of the dsRNA adjuvant was driven by a CMV promoter in ND1.1 and by an SV40 promoter in VXA-H5-02.

Recombinant Ad5 particles were generated by transfection of the respective vector plasmids into Expi293F Inducible cells (Thermo Fisher Scientific) and were subsequently purified by cesium chloride density-gradient ultracentrifugation. Infectious titers of purified rAd5 preparations were determined using a modified hexon immunostaining assay, similar to the method described by Hickey et al. (46). To determine the infectious units (IU), HEK293 cells were infected with serial dilutions of each vector and incubated to permit expression of adenoviral hexon protein. Infected cells were subsequently fixed and immunostained using an anti-hexon antibody (Abcam), and hexon-positive cells were enumerated. Infectious titers were calculated from the number of hexon-positive cells at countable vector dilutions and expressed as infectious units per milliliter (IU/mL).

### 4.2 HIEC-6 adenovirus transduction

HIEC-6 human intestinal epithelial cells (ATCC) were cultured in Opti-MEM I supplemented with 4% FBS, GlutaMax-L, 1% penicillin/streptomycin, 20 mM HEPES, 10 ng/ml human epidermal growth factor with 5% CO_2_ until ~ 80-90% confluence. 12-well tissue culture plates were seeded at 3.7x10^5^ cells/well and infected with an MOI of 50, 100 or 200 in two independent experiments. At 42 hours post-infection, cells were harvested with TrypLE and stained with a fixable violet viability dye at 1:500 in PBS (Invitrogen). Hemagglutinin surface expression was detected using CR9114 monoclonal antibody (Creative Biolabs) at 1:400 in PBS, 2% FBS, 25mM EDTA, followed by a PE-conjugated anti-human IgG antibody at 1:100 (Invitrogen). The samples were measured using the Attune instrument (Thermo Fisher). Data was analyzed using FlowJo (Treestar) and Prism (GraphPad Software version 10).

### 4.3 Mouse immunization study

Murine studies were reviewed and approved by the Institutional Animal Care and Use Committee (IACUC) at Mispro Biotech Services. Female 8-10-weeks old BALB/c mice (Jackson Labs) were anesthetized with isoflurane, then intranasally immunized with PBS or 1x10^5^ IU rAd5-H5 vaccines on Day 0 and Day 28. Five mice were bled by submandibular bleeding on Day -1, Day 14, and Day 27, and by terminal cardiac puncture on Day 42. Blood was collected in sterile microcentrifuge tubes or serum separator tubes (BD Biosciences) and allowed to clot at RT for at least 30 minutes, then was centrifuged for 5 mins at 15,000 rpm. Sera were aliquoted and stored at -80 °C.

### 4.4 Ferret immunization and challenge study

Ferret studies were reviewed and approved by the IACUC at Southern Research.

Male and female 6–8-month-old ferrets (*Mustela putorius*) that were seronegative for H1N1, H3N2, Victoria, Yamagata, and H5N1 (A/dairy cattle/Texas/24-008749-002-v/2024) were fasted for 2–4 hours prior to vaccination. To perform peroral endoscopic delivery, ferrets were anesthetized with ketamine/xylazine/atropine (KXA) by injection to the inguinal area, intubated, and maintained with an isoflurane/O2 mixture. A bronchoscope was then gently inserted through the oral cavity, esophagus, esophageal sphincter, and stomach to the proximal small intestine. 5 ml PBS or 1 x 10^10^ IU rAd5-H5 vaccines were then delivered to the small intestine on Day 0 and Day 28. Blood was collected from the ferrets on Day 0, Day 28, and Day 52 of vaccination into serum separator tubes. Blood was allowed to clot at RT for at least 30 minutes, then was centrifuged for 5 mins at 15,000 rpm. Sera were aliquoted and stored at -80°C.

Nasal wash samples were obtained by anesthetizing the ferrets with KXA, followed by flushing the nostril with 1 ml nasal wash buffer using a syringe and catheter. Nasal wash fluids obtained were aliquoted and stored at -80°C.

Ferrets were challenged on Day 56 with 1x10^5^ TCID_50_/ml of H5N1, clade 2.3.4.4b virus, strain A/dairy cattle/Texas/24-008749-002-v/2024 (GISAID accession: EPI_ISL_19032063) in the Animal Biosafety Level 3 facility in Southern Research. Briefly, ferrets were anesthetized with KXA, then 0.5 ml of virus was administered to each nasal cavity (total 1 ml) in a drop-wise manner to ensure full inhalation of the inoculum. Following administration, the animal was held in an upright position for approximately 30 seconds. After infection, ferrets were monitored for body weight, temperature, and clinical signs of infection for up to 9 days post infection.

Activity and clinical observations were recorded daily on Days 0, 28, and 55–65. Ferrets were anesthetized with ketamine, xylazine, and atropine by inguinal injection on Days 0, 28, and 55–65. Body weight was recorded daily on Days 0, 28, and 55–65, and body temperature was measured daily by microchip from Days 55–65. For animals meeting moribund criteria, body weight, body temperature, and clinical observations were recorded before euthanasia. The number of clinical signs was calculated for each animal independent of severity. Activity was scored daily from Days 56–65 using the following scale: 0, alert, playful, and active; 1, alert but playful or active only when stimulated; 2, alert but not playful or active when stimulated; and 3, neither alert nor playful or active when stimulated. Activity-score changes preceded clinical signs by approximately one day. On Day 65, all remaining animals were euthanized by vena cava or intracardiac injection of a barbiturate overdose at a minimum dose of 100 mg/kg.

### 4.5 Anti-HA antibody binding assay

Avi-tagged recombinant H5 hemagglutinin proteins from A/Indonesia/5/2005, clade 2.1.3.2 (SinoBiological), A/Vietnam/1203/2004, clade 1 (SinoBiological), or biotinylated A/Greylag Goose/Scotland/024915/2022, clade 2.3.4.4b (Native Antigen Company) were coated on Mesoscale Discovery 4-spot U-plex plates (MSD) overnight at 4 °C. The plates were then blocked with 3% blocking buffer (Amersham ECL Blocking Agent, Cytvia) for 1 hour at RT, washed with PBST (PBS + 0.05% tween 20), and incubated with serum or nasal wash fluids (NWF) serially diluted to the indicated dilution with 1% blocking buffer for 1 hour at RT. Bound HA-specific antibodies were detected using 1 μg/ml of either sulfo-tagged conjugated goat anti-mouse IgG (MSD), in-house sulfo-tagged conjugated goat anti-ferret IgG (H+L) (Fortis Life Sciences), or in-house sulfo-tagged conjugated goat anti-dog IgA (Bio-Rad), which cross-reacts to ferret IgA for 1 hour at RT. Plates were washed with PBST and read on a Meso QuickPlex SQ 120 instrument (MSD) and reported as RLU values and fold change over Day -1 or 0.

### 4.6 Total IgG and IgA measurement

NWF samples were normalized by total IgG and IgA in the sample. To measure total IgG and IgA, biotinylated goat anti-ferret IgG (H+L) and biotinylated goat anti-dog IgA were coated on Mesoscale Discovery 2-spot U-plex plates (MSD) overnight at 4C. The plates were then blocked with 3% blocking buffer (Amersham ECL Blocking Agent, Cytvia) for 1 hour at RT, washed with PBST, and incubated with NWF serially diluted to the indicated dilution with 1% blocking buffer for 1 hour at RT. Bound IgG or IgA antibodies were detected using 1 μg/ml of sulfo-tagged conjugated goat anti-ferret IgG (H+L) (Fortis Life Sciences), which recognized both ferret IgG and IgA, for 1 hour at RT. Plates were washed with PBST and read on a Meso QuickPlex SQ 120 instrument (MSD) and reported as RLU values. Final RLU values for specific and total antibody measurements were adjusted based on the respective dilutions and then reported as specific RLU/total RLU and fold change over Day 0.

### 4.7 Pseudovirus neutralization

Pseudovirus neutralization was performed using 96-well rapid pseudoviral neutralization kit (Virongy Biosciences). Briefly, heat-inactivated serum or NWF from ferrets collected on Day 52 post-immunization was incubated with clade 2.3.4.4b (A/Nevada/10/2025) or clade 2.3.2.1e (A/Cambodia/25030237/2025) alpha-pseudovirus particles at RT for 30 minutes before adding onto ready-to-use target cells for overnight incubation at 37 °C, 5% CO_2_. On the next day, the cells were lysed and luciferase signal was read on the Biotek Cytation 7 plate reader (Agilent) by measuring luminescence. % infection was calculated using the formula: (Sample – cell only)/(Infection control – cell only) * 100%. Neutralizing titer 50 was reported as the reciprocal of dilution that generated 50% neutralization.

### 4.8 Quantitative RT-qPCR

NWF collected after clade 2.3.4.4b challenges on days 1, 3, 5, 7, 9 post infection were performed in the BSL3 laboratory in Southern Research. Some samples were collected on Day 6 or Day 8 from moribund animals and were included in Day 7 and Day 9, respectively, for analysis. Viral RNA from NWF was isolated using RNeasy mini kit (Qiagen), quantified by nanophotometer, and stored at – 80 °C. 50 ng - 2 μg of total RNA was used to generate cDNA using oligo-dT primer and QuantiTect Reverse Transcription kit (Qiagen). qPCR was then performed using primers based on the WHO Matrix Influenza Prime Time Assay, the Taqman Fast Virus 1-Step Master Mix (Thermo Fisher), and the QuantStudio 6 Flex instrument (Thermo Fisher). For internal amplification control, VetMax Xeno Internal Positive Control (Thermo Fisher) was used in all samples to monitor for qPCR inhibition. A standard curve was determined using synthetic DNA for Matrix protein (IDT) generated to determine viral copies in each sample. Forward primer CTTCTAACCGAGGTCGAAACGTA, reverse primer GGTGACAGGATTGGTCTTGTCTTTA, taqman probe 56-FAM/TCAGGCCCC/ZEN/CTCAAAGCCGAG/3IABkFQ/.

### 4.9 TCID_50_

NWF samples that were positive by RT-qPCR were further evaluated by TCID_50_. NWF samples were thawed and centrifuged at 1,100 - 1,500 RPM for 10 minutes at 2 - 8 °C to isolate the supernatant. 50 μL of supernatant was added to 450 μL of infection media to achieve an initial 1:10 dilution and then serially diluted 10-fold for up to 7 dilutions. Four replicates of each dilution (100 μL/well) were added to 96-well plates seeded with MDCK cells. Each sample/replicate was then incubated for 72 hours. Cytopathic effects were quantified by Cell-titer-Glo (Promega) to determine TCID_50_ titer using the Reed-Muench method. Negative PCR samples were reported as 0 for TCID_50_ for analysis.

### 4.10 Statistical analyses

Analyses of flow cytometric data were performed using FlowJo (TreeStar Software). Statistical significance was determined using Prism (GraphPad Software version 10). For murine and ferret immunogenicity, 2-way ANOVAs were conducted with Sidak or Dunnett multiple comparison post-tests, depending on the number of animals per group. Survival after challenge was analyzed by Kaplan-Meier survival analysis, with group comparisons performed using the log-rank (Mantel-Cox) test and Holm-Sidak correction for multiple comparisons. Fisher’s exact tests were used to compare survival proportions, with the odds ratios and 95% confidence intervals reported.

## Data Availability

The original contributions presented in the study are included in the article/**Supplementary Material**. Further inquiries can be directed to the corresponding author.

## References

[1] Xie Z Yang J Jiao W Li X Iqbal M Liao M . Clade 2.3.4.4b highly pathogenic avian influenza H5N1 viruses: knowns, unknowns, and challenges. J Virol. (2025) 99:e0042425. doi: 10.1128/jvi.00424-25 40340397 PMC12172449

[2] CDC . H5 Bird Flu: Current Situation. Atlanta, GA: United States Center for Disease Control And Prevention (2025).

[3] FDA . Vaccines Licensed for Use in the United States. Silver Spring, MD: United States Food and Drug Administration (2025).

[4] Khurana S King LR Manischewitz J Posadas O Mishra AK Liu D . Licensed H5N1 vaccines generate cross-neutralizing antibodies against highly pathogenic H5N1 clade 2.3.4.4b influenza virus. Nat Med. (2024) 30:2771–6. doi: 10.1038/s41591-024-03189-y 39013430

[5] Hawman DW Tipih T Hodge E Stone ET Warner N McCarthy N . Clade 2.3.4.4b but not historical clade 1 HA replicating RNA vaccine protects against bovine H5N1 challenge in mice. Nat Commun. (2025) 16:655. doi: 10.1038/s41467-024-55546-7 39809744 PMC11732985

[6] Lavelle EC Ward RW . Mucosal vaccines - fortifying the frontiers. Nat Rev Immunol. (2022) 22:236–50. doi: 10.1038/s41577-021-00583-2 PMC8312369 34312520

[7] Krammer F . The human antibody response to influenza A virus infection and vaccination. Nat Rev Immunol. (2019) 19:383–97. doi: 10.1038/s41577-019-0143-6 30837674

[8] Ying B Pyles K Darling TL Seehra K Pham T Huang LC . An intranasal adenoviral-vectored vaccine protects against highly pathogenic avian influenza H5N1 in naive and antigen-experienced animals. Cell Rep Med. (2026) 7:102582. doi: 10.1016/j.xcrm.2025.102582 41619721 PMC12923973

[9] Hatta M Brock N Hauguel T Feng C Huang Y Ritter JM . Influenza mRNA vaccine reduces pathogenicity and transmission of A(H5N1) virus in a ferret model. NPJ Vaccines. (2025) 10:263. doi: 10.1038/s41541-025-01318-3 41318623 PMC12728207

[10] Furey C Scher G Ye N Kercher L DeBeauchamp J Crumpton JC . Development of a nucleoside-modified mRNA vaccine against clade 2.3.4.4b H5 highly pathogenic avian influenza virus. Nat Commun. (2024) 15:4350. doi: 10.1038/s41467-024-48555-z 38782954 PMC11116520

[11] Flitter BA Braun MR Tucker SN . Drop the needle; A temperature stable oral tablet vaccine is protective against respiratory viral pathogens. Vaccines (Basel). (2022) 10:593. doi: 10.3390/vaccines10040593 35455342 PMC9031097

[12] World Health Organization . WHO preferred product characteristics for next generation influenza vaccines, second edition. (2025).

[13] Kim L Martinez CJ Hodgson KA Trager GR Brandl JR Sandefer EP . Systemic and mucosal immune responses following oral adenoviral delivery of influenza vaccine to the human intestine by radio controlled capsule. Sci Rep. (2016) 6:37295. doi: 10.1038/srep37295 27881837 PMC5121599

[14] Liebowitz D Gottlieb K Kolhatkar NS Garg SJ Asher JM Nazareno J . Efficacy, immunogenicity, and safety of an oral influenza vaccine: a placebo-controlled and active-controlled phase 2 human challenge study. Lancet Infect Dis. (2020) 20:435–44. doi: 10.1016/s1473-3099(19)30584-5 31978354

[15] McIlwain DR Chen H Rahil Z Bidoki NH Jiang S Bjornson Z . Human influenza virus challenge identifies cellular correlates of protection for oral vaccination. Cell Host Microbe. (2021) 29:1828–37:e5. doi: 10.1016/j.chom.2021.10.009 34784508 PMC8665113

[16] Liebowitz D Lindbloom JD Brandl JR Garg SJ Tucker SN . High titre neutralising antibodies to influenza after oral tablet immunisation: a phase 1, randomised, placebo-controlled trial. Lancet Infect Dis. (2015) 15:1041–8. doi: 10.1016/s1473-3099(15)00266-2 26333337

[17] Dunning J Thwaites RS Openshaw PJM . Seasonal and pandemic influenza: 100 years of progress, still much to learn. Mucosal Immunol. (2020) 13:566–73. doi: 10.1038/s41385-020-0287-5 32317736 PMC7223327

[18] Li J Wu G Huang Z Hu J Tie X Wu H . Advances and prospects of respiratory mucosal vaccines: mechanisms, technologies, and clinical applications. NPJ Vaccines. (2025) 10:230. doi: 10.1038/s41541-025-01280-0 41224752 PMC12612081

[19] Gould VMW Francis JN Anderson KJ Georges B Cope AV Tregoning JS . Nasal igA provides protection against human influenza challenge in volunteers with low serum influenza antibody titre. Front Microbiol. (2017) 8:900. doi: 10.3389/fmicb.2017.00900 28567036 PMC5434144

[20] Zhang X Zhang J Chen S He Q Bai Y Liu J . Progress and challenges in the clinical evaluation of immune responses to respiratory mucosal vaccines. Expert Rev Vaccines. (2024) 23:362–70. doi: 10.1080/14760584.2024.2326094 38444382

[21] Russell MW Moldoveanu Z Ogra PL Mestecky J . Mucosal immunity in COVID-19: A neglected but critical aspect of SARS-coV-2 infection. Front Immunol. (2020) 11:611337. doi: 10.3389/fimmu.2020.611337 33329607 PMC7733922

[22] Scallan CD Tingley DW Lindbloom JD Toomey JS Tucker SN . An adenovirus-based vaccine with a double-stranded RNA adjuvant protects mice and ferrets against H5N1 avian influenza in oral delivery models. Clin Vaccine Immunol. (2013) 20:85–94. doi: 10.1128/cvi.00552-12 23155123 PMC3535765

[23] Peters W Brandl JR Lindbloom JD Martinez CJ Scallan CD Trager GR . Oral administration of an adenovirus vector encoding both an avian influenza A hemagglutinin and a TLR3 ligand induces antigen specific granzyme B and IFN-gamma T cell responses in humans. Vaccine. (2013) 31:1752–8. doi: 10.1016/j.vaccine.2013.01.023 23357198

[24] Vaxart . Vaxart reports positive clinical data demonstrating that its second-generation vaccine technology produces much stronger antibody responses than its first-generation technology. (2025).

[25] Scallan CD Lindbloom JD Tucker SN . Oral modeling of an adenovirus-based quadrivalent influenza vaccine in ferrets and mice. Infect Dis Ther. (2016) 5:165–83. doi: 10.1007/s40121-016-0108-z 27071663 PMC4929087

[26] Beukenhorst AL Frallicciardi J Rice KL Koldijk MH Moreira de Mello JC Klap JM . A pan-influenza monoclonal antibody neutralizes H5 strains and prophylactically protects through intranasal administration. Sci Rep. (2024) 14:3818. doi: 10.1038/s41598-024-53049-5 38360813 PMC10869794

[27] Belser JA Katz JM Tumpey TM . The ferret as a model organism to study influenza A virus infection. Dis Model Mech. (2011) 4:575–9. doi: 10.1242/dmm.007823 21810904 PMC3180220

[28] World Health Organization . (2025). Disease Outbreak News; Avian Influenza A (H5N1) in Cambodia. Available online at: https://www.who.int/emergencies/disease-outbreak-news/item/2024-DON575 (Accessed July 5, 2025).

[29] Siegers JY Xie R Byrne AMP Edwards KM Hu S Yann S . Resurgence of zoonotic highly pathogenic avian influenza A (H5N1) virus in Cambodia. N Engl J Med. (2025) 393:1650–2. doi: 10.1056/NEJMc2504302 PMC12614772 41124637

[30] Yang H Carney PJ Mishin VP Guo Z Chang JC Wentworth DE . Molecular characterizations of surface proteins hemagglutinin and neuraminidase from recent H5Nx avian influenza viruses. J Virol. (2016) 90:5770–84. doi: 10.1128/jvi.00180-16 27053557 PMC4886766

[31] Smith RE Choudhary S Ramirez JA . Ferrets as models for viral respiratory disease. Comp Med. (2023) 73:187–93. doi: 10.30802/aalas-cm-22-000064 37258084 PMC10290486

[32] Reuman PD Keely S Schiff GM . Assessment of signs of influenza illness in the ferret model. J Virol Methods. (1989) 24:27–34. doi: 10.1016/0166-0934(89)90004-9 2760163

[33] Cox RJ . Correlates of protection to influenza virus, where do we go from here? Hum Vaccin Immunother. (2013) 9:405–8. doi: 10.4161/hv.22908 23291930 PMC3859764

[34] Hatta M Hatta Y Choi A Hossain J Feng C Keller MW . An influenza mRNA vaccine protects ferrets from lethal infection with highly pathogenic avian influenza A(H5N1) virus. Sci Transl Med. (2024) 16:eads1273. doi: 10.1126/scitranslmed.ads1273 39693411 PMC12100637

[35] Liu Y Deng S Ren S Tam RC Liu S Zhang AJ . Intranasal influenza virus-vectored vaccine offers protection against clade 2.3.4.4b H5N1 infection in small animal models. Nat Commun. (2025) 16:3133. doi: 10.1038/s41467-025-58504-z 40169649 PMC11962148

[36] Moderna . Moderna announces update on investigational pandemic influenza program. (2025).

[37] Muramatsu M Yoshida R Yokoyama A Miyamoto H Kajihara M Maruyama J . Comparison of antiviral activity between IgA and IgG specific to influenza virus hemagglutinin: increased potential of IgA for heterosubtypic immunity. PloS One. (2014) 9:e85582. doi: 10.1371/journal.pone.0085582 24465606 PMC3895000

[38] Corthesy B . Multi-faceted functions of secretory IgA at mucosal surfaces. Front Immunol. (2013) 4:185. doi: 10.3389/fimmu.2013.00185 PMC3709412 23874333

[39] Dai M Xu C Chen W Liao M . Progress on chicken T cell immunity to viruses. Cell Mol Life Sci. (2019) 76:2779–88. doi: 10.1007/s00018-019-03117-1 31101935 PMC11105491

[40] Janssens Y Joye J Waerlop G Clement F Leroux-Roels G Leroux-Roels I . The role of cell-mediated immunity against influenza and its implications for vaccine evaluation. Front Immunol. (2022) 13:959379. doi: 10.3389/fimmu.2022.959379 36052083 PMC9424642

[41] Wang Z Lorenzi JCC Muecksch F Finkin S Viant C Gaebler C . Enhanced SARS-CoV-2 neutralization by dimeric IgA. Sci Transl Med. (2021) 13:eabf1555. doi: 10.1126/scitranslmed.abf1555 33288661 PMC7857415

[42] Deming ME Toapanta FR Pasetti M Golding H Khurana S Hamouda T . An intranasal adjuvanted, recombinant influenza A/H5 vaccine primes against diverse H5N1 clades: a phase I trial. Nat Commun. (2025) 16:9321. doi: 10.1038/s41467-025-64686-3 41198655 PMC12592354

[43] Langel SN Johnson S Martinez CI Tedjakusuma SN Peinovich N Dora EG . Adenovirus type 5 SARS-CoV-2 vaccines delivered orally or intranasally reduced disease severity and transmission in a hamster model. Sci Transl Med. (2022) 14:eabn6868. doi: 10.1126/scitranslmed.abn6868 35511920 PMC9097881

[44] Flitter BA Gillard J Greco SN Apkarian MD D'Amato NP Nguyen LQ . An oral norovirus vaccine generates mucosal immunity and reduces viral shedding in a phase 2 placebo-controlled challenge study. Sci Transl Med. (2025) 17:eadh9906. doi: 10.1126/scitranslmed.adh9906 40367195

[45] van de Ven K Lanfermeijer J van Dijken H Muramatsu H Vilas Boas de Melo C Lenz S . A universal influenza mRNA vaccine candidate boosts T cell responses and reduces zoonotic influenza virus disease in ferrets. Sci Adv. (2022) 8:eadc9937. doi: 10.1126/sciadv.adc9937 36516261 PMC9750153

[46] Hickey JM Jacob SI Tait AS Vahid FD Barritt J Rouse S . Measurement of adenovirus-based vector heterogeneity. J Pharm Sci. (2023) 112:974–86. doi: 10.1016/j.xphs.2022.12.012 36563855 PMC9767660

