# Supplementary material for: An adenovirus-based mucosal vaccine induces cross-neutralizing antibodies and protects against lethal H5N1 infection in ferrets
Source: Front Immunol. 2026 Oct 8;17:1863888. doi: 10.3389/fimmu.2026.1863888 (PMC13687837; doi:10.3389/fimmu.2026.1863888)
Supplement: Supplementary file 1 [file DataSheet1.docx]

Supplementary Material

**Supplementary Figure 1. Anti-H5 cross reactive serum IgG antibodies elicited in mice immunized with ND1.1 or VXA-H5-02 via intranasal vaccination.**

**A)** Schematic of mouse immunization. Female 8-10-week-old BALB/c mice were immunized via the intranasal route with 1 x 10^5^ IU of ND1.1 or VXA-H5-02 on Day 0 and 28. Serum samples were collected on Day -1, 14, 27, and 42 to measure IgG antibody response by MSD. **B)** RLU and **C**) fold rise of anti-clade 2.3.4.4b HA (left), anti-clade 2.1.3.2 HA (middle), and anti-clade 1 HA (right) of ND1.1 or VXA-H5-02 immunized mice. Graphs represent the average of 5 mice per group +/- S.E.M. Statistical analysis was performed using a 2-way ANOVA with Sidak’s multiple comparison test. * p < 0.05, *** p < 0.001, **** p < 0.0001.

**Supplementary Figure 2. RLU of cross-reactive anti-H5 antibodies from periorally vaccinated ferrets. A-D)** RLU of serum IgA **(A)**, serum IgG **(B)**, nasal wash IgA **(C)**, nasal wash IgG **(D)** of anti-Clade 2.3.4.4b HA (left), anti-Clade 2.1.3.2 HA (middle), and anti-Clade 1 HA (right) of ND1.1 or VXA-H5-02 immunized ferrets. Graphs representing the average of 8 ferrets (4M/4F) per group +/- S.E.M. Statistical analysis was performed using a 2-way ANOVA with Dunnett’s multiple comparison test. * p < 0.05, **, p < 0.01, *** p < 0.001, **** p < 0.0001.

**Supplementary Figure 3. Ferrets orally vaccinated with VXA-H5-02 produce neutralizing antibodies in NWF. A)** Serial dilution of PBS, ND1.1, or VXA-H5-02 nasal wash fluid neutralized alpha-pseudovirus for Clade 2.3.4.4b virus (Genotype D, A/Nevada/10/2025). The % infected cells w determined by measuring the luminescence at 20 hours post infection. Graph represents the average of 8 ferrets per group +/- S.E.M. **(B)** Number of ferrets with undiluted NWF exhibiting less than 50% infected cells (orange) or greater than 50% infected cells (gray).

**Supplementary Figure 4.** **Protein sequence alignment of Clade 2.3.2.1e, Clade 2.3.4.4b, Clade 2.1.3.2, and Clade 1 HA sequences.** Protein sequence alignment of the HA protein from Clade 2.3.4.4b (represented by the VXA-H5-02 transgene), Clade 2.1.3.2 (represented by the ND1.1 transgene), Clade 2.3.2.1e (represented by A/Cambodia/25030237/2025), and Clade 1 (represented by A/Vietnam/1203/2004) using the Clustal Omega multiple sequence alignment algorithm (Uniprot.org). H1 antigenic regions: (1) Sa, (2) Sb, (3) Ca, (4) Cb. H3 antigenic regions: A, B, C, D, E. S: signal sequence; 1: HA1; 2: HA2; T: Transmembrane domain; I: Intracellular domain.

**Supplementary Figure 5. Summary of clinical symptoms after a Clade 2.3.4.4b challenge in vaccinated and PBS control ferrets. A)** % Body weight loss, **B)** body temperature, **C)** Overall number of clinical symptoms (i.e. dehydration, diarrhea, nasal discharges, hypoactivity, sneezing, rough coat, abnormal breathing) of ferrets post challenge. The number of clinical symptoms that were recorded were added up per animal independent of the severity of the observation. Each thin line represents an individual ferret. Circles and thick line represent the averaged value of the indicated parameter +/- S.E.M.

**Supplementary Figure 6. Clinical Symptoms Following Challenge in Individual Ferrets.**  All clinical symptoms observed for individual ferrets are reported by day post challenge. Animal number and sex for each ferret designated by M or F is shown. Animals that were found to be moribund or succumbed to infection are designated with a pink box.
